# O099: Making a training CD-ROM on activity waste at risk care

**DOI:** 10.1186/2047-2994-2-S1-O99

**Published:** 2013-06-20

**Authors:** B Chardon

**Affiliations:** 1 CEDDES, Montpellier, France

## Introduction

Within the context of a mission of the regional Health/Environment departments, CEDDES produced a second training CD-ROM in november 2010, about hazardous health care waste : infectious, chemical, toxic, radioactive waste.

## Objectives

To respond to needs for training the different categories of staff in health care , education establishments, research centres in human medicine and veterinary, and professionals of the environment, in order to set up an optimal health care waste management.

## Methods

Stages :

- Selection of the Steering Committee - period of the mission : 18 months

- Document retrieval

- Writing

## Results

Production of a CD-rom of 400 screens divided in 5 chapters: HCW, infectious HCW, chemical, toxic HCW, radioactive HCW, general waste; illustrated with 300 photos; glossary of 250 words; repertoire of 240 acronyms; proposals for evaluation quiz; selection of websites.

## Conclusion

The CD-rom can be used in developed countries as well as developing ones.

A suitable HCWM is a complex system with regulatory, organizational, structural, budgetary components; with sanitary, environmental, economic, legal implications.

Key elements: - Hand hygiene, control of health care associated infections, in the context of the new world stakes of patient safety; - Management of sharps and AES control.

The CD-rom is a teaching aid to set up a long-lasting change of behaviour towards a common culture of safety. It got the Prize 2012 of the STHSS.

## Disclosure of interest

None declared.

